# Supplementary material for: G2M Cell Cycle Pathway Score as a Prognostic Biomarker of Metastasis in Estrogen Receptor (ER)-Positive Breast Cancer
Source: Int J Mol Sci. 2020 Apr 22;21(8):2921. doi: 10.3390/ijms21082921 (PMC7215898; doi:10.3390/ijms21082921)
Supplement: Supplementary file 1 [file ijms-21-02921-s001.pdf]

G2M Cell Cycle Pathway Score as a Prognostic Biomarker of Metastasis in Estrogen Receptor (ER)-Positive Breast Cancer

Masanori Oshi, Hideo Takahashi, Yoshihisa Tokumaru, Li Yan, Omar M. Rashid, Ryusei Matsuyama, Itaru Endo and Kazuaki Takabe

Table S1. Genes that overlaps with G2M and other pathway gene sets.

Gene set name	Total genes	Genes in overlap
HALLMARK_G2M_CHECKPOINT	200	200
HALLMARK_E2F_TARGETS	200	73 (36.5%)
HALLMARK_MITOTIC_SPINDLE	199	39 (19.6%)
HALLMARK_MYC_TARGETS_V1	200	29 (14.5%)
HALLMARK_MYC_TARGETS_V2	58	6 (10.3%)
HALLMARK_UNFOLDED_PROTEIN_RESPONSE	113	6 (5.3%)
HALLMARK_MTORC1_SIGNALING	200	9 (4.5%)
HALLMARK_PI3K_AKT_MTOR_SIGNALING	105	4 (3.8%)
HALLMARK_DNA_REPAIR	150	2 (1.3%)

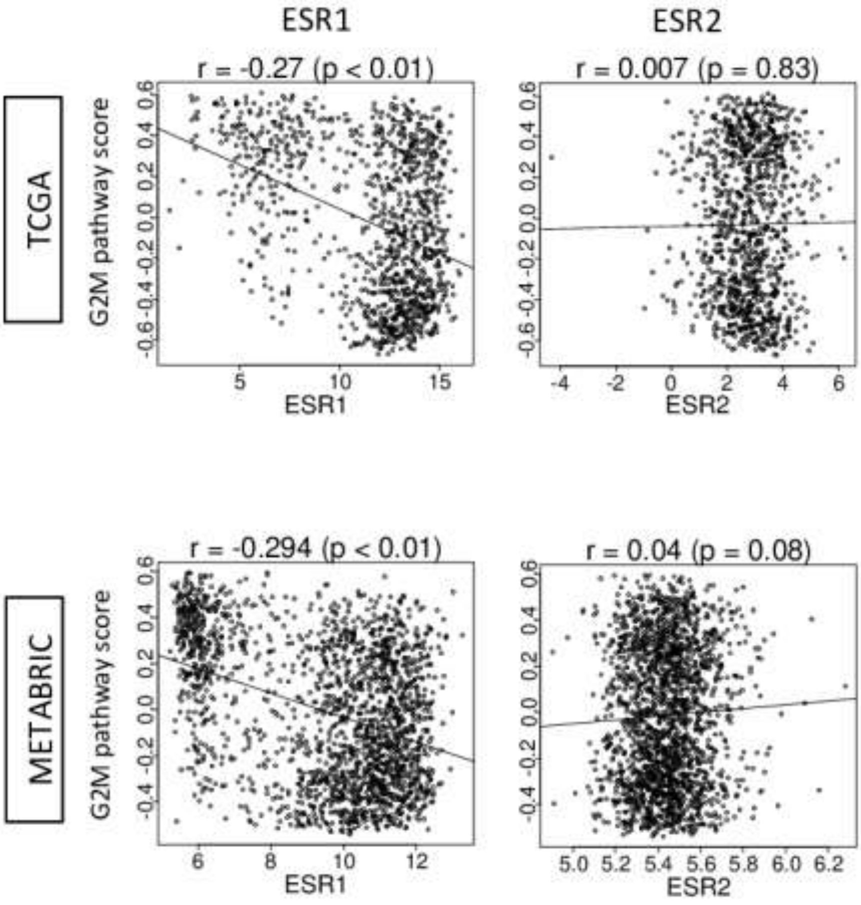

Figure S1. Correlation between tumor G2M pathway score and ESR1/2 gene expression. Correlation plot between the G2M score and ESR1/2 gene expression with Spearman's rank correlation coefficient and p-value in TCGA and METABRIC cohorts. ESR, estrogen receptor.

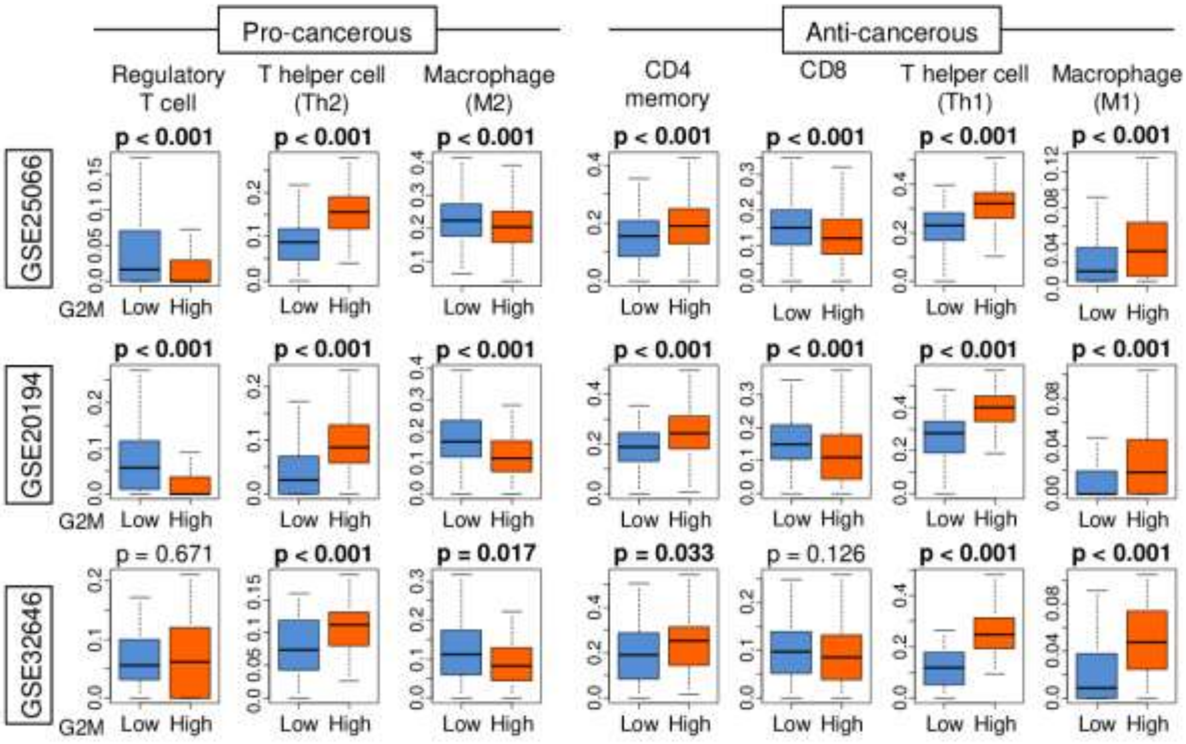

Figure S2. Immune features of G2M activity-high and -low primary breast cancer tumors of multiple neoadjuvant chemotherapy treatment cohorts. Tukey boxplots are shown for each cohort for features quantified from bulk tumor gene expression using the xCell method. The G2M score was calculated from tumor gene expression as the single-sample gene set variation analysis score for the Hallmark G2M gene set, and within-cohort median value was used to identify tumors with high and low scores. Depicted P values are calculated using one-way ANOVA test.

.

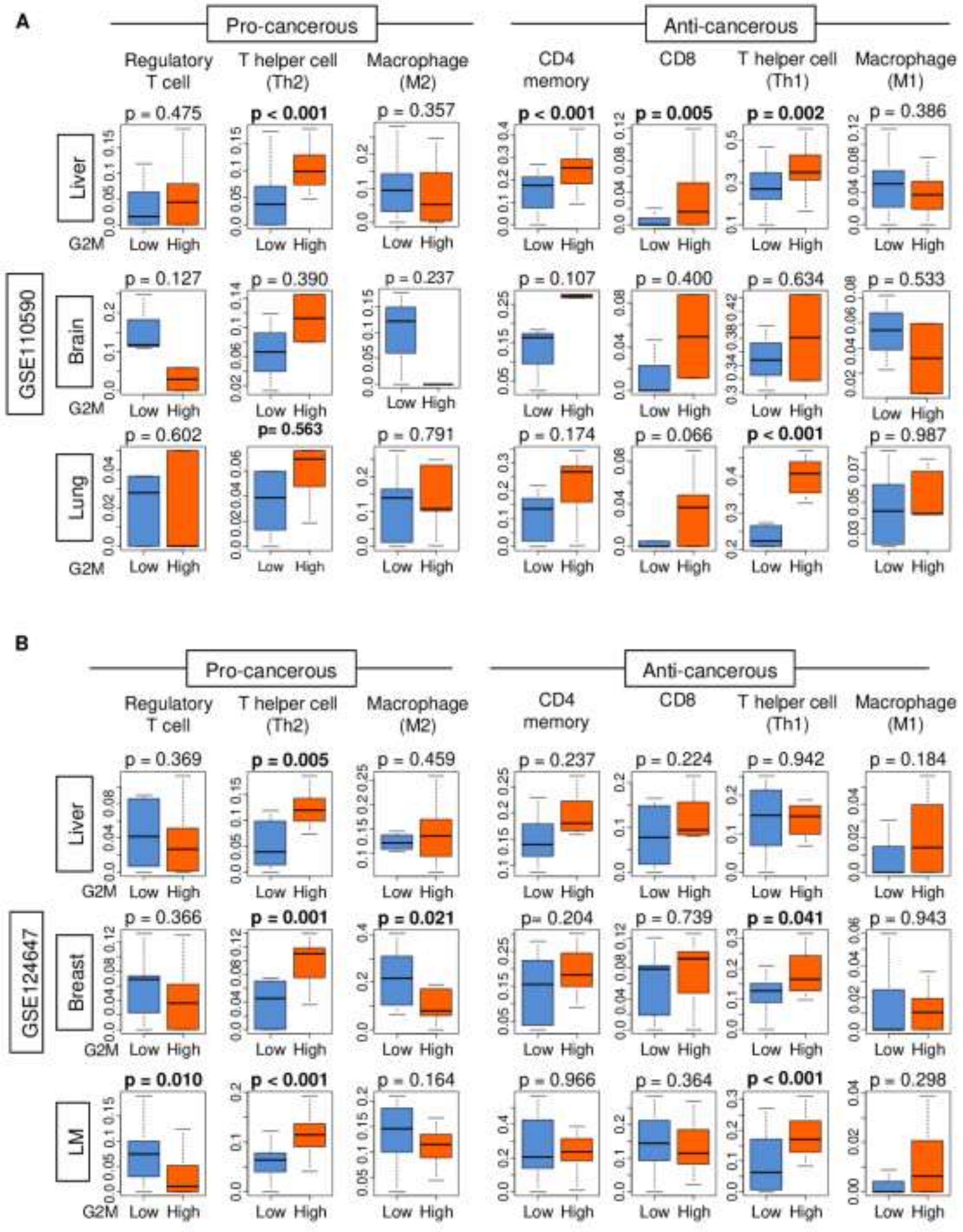

Figure S3. Immune features of G2M activity-high and -low breast cancer metastasis tumors. Tukey boxplots are shown for features quantified from gene expression of metastasis lesions using the xCell method. The G2M score was calculated from tumor gene expression as the single-sample gene set variation analysis score for the Hallmark G2M gene set, and within-cohort median value was used to identify tumors with high and low scores. Depicted P values are calculated using one-way ANOVA test.
